# Awareness and implementation of the time-out procedure among surgical teams: current status and influencing factors

**DOI:** 10.3389/fpubh.2026.1850411

**Published:** 2026-06-12

**Authors:** Zheng Liu, Xiaoping Luo, Xiaozu Liao, Zhihong Zhang

**Affiliations:** Department of Anesthesiology, Zhongshan City People’s Hospital, Zhongshan, Guangdong, China

**Keywords:** cognitive, influencing factors, performance, surgical team, time-out procedure

## Abstract

**Background:**

In China, the Time-out procedure has been officially promoted and required for clinical use since 2010. Nevertheless, the overall cognition and practical compliance of surgical staff still need to be improved, and relevant influencing factors have not been fully clarified. Therefore, this study was conducted to investigate the current status of cognition and implementation of the Time-out procedure among surgical team members, and further analyze its related influencing factors.

**Methods:**

A structured questionnaire comprising three sections was administered: a general demographic survey, a self-developed instrument to assess awareness and implementation of the time-out procedure, and an operating room safety culture scale. The self-developed questionnaire underwent expert review and a preliminary validation study (Cronbach’s *α* = 0.817) to confirm its internal consistency. Sample size was determined based on multiple regression requirements, ensuring at least 10 subjects per independent variable with an expected non-response rate accounted for.

**Primary and secondary evaluation indicators:**

*Primary outcome*: time-out implementation. *Secondary indicators*: Individual cognition; professional knowledge; safety culture.

**Results:**

The score of the Time-out Procedure Knowledge Test was 3.80 ± 0.90, total cognitive score of the Time-out procedure was 72.95 ± 14.82. Occupation, participation in Time-out team training, and operating room safety culture were the main factors affecting the perception of the Time-out procedure (*p* < 0.05). In addition, occupation, working period, Time-out initiator title, the completeness of the Time-out procedure, supervision and management system, and hospital safety atmosphere were the main factors influencing the implementation rate (*p* < 0.05).

**Conclusion:**

The results showed that surgical team members had moderate cognition regarding the Time-out procedure, yet their execution scores and implementation levels were comparatively low. This study also distinguished different influencing factors for cognition and implementation. Subsequent research could adopt stratified random sampling and carry out multi-center studies, together with objective observation to minimize self-report bias and improve the generalizability of research findings.

## 1 Introduction

With the rapid development of medical technology, the number of operations in major hospitals has increased steadily, while the incidence of surgical adverse events has also risen. These adverse events impose significant burdens on patients, healthcare professionals, and society (1, 2). Notably, previous studies have demonstrated that surgical safety verification can reduce adverse events by more than 50% (2). In 2003, the United States Joint Commission introduced the Time-out procedure to enhance patient safety and reduce surgical adverse events. This procedure requires all surgical team members to verify critical patient information before incision to ensure correct patient identity, surgical site, and procedure, thereby ensuring the accuracy of the surgical site, procedure, and patient identity. It has become a powerful tool for establishing an operating room safety culture (3, 4). In 2010, the Ministry of Health of China mandated the implementation of a surgical safety verification system, including the Time-out procedure, across major hospitals nationwide (5). Despite these efforts, the awareness and application of the Time-out procedure among Chinese surgical team members remain relatively low. Furthermore, the verification process in the operating room, typically carried out by three parties, has not been fully standardized, Failure to rigorously implement the verification protocol may result in adverse events including wrong-site surgery, wrong-patient operations and other intraoperative errors. Such incidents severely compromise patient safety and further elevate overall clinical medical risks. Improving surgical team members’ understanding and active execution of the Time-out procedure to reduce adverse events and ensure patient safety is a critical issue in operating room research. Since initiating the procedure requires effective communication and cooperation among surgeons, anesthesiologists, and nurses, discrepancies in their understanding and safety culture can hinder proper execution. However, despite clearly identified gaps in both awareness and implementation, studies exploring these issues within the Chinese healthcare context are limited. Existing research predominantly focuses on narrower aspects, such as operating room nurses’ compliance (4). Moreover, there has been minimal examination of the cultural and operational barriers unique to the implementation of the Time-out procedure in China, leaving substantial gaps in our understanding of its practical application and overall execution quality. Recent international research further underscores the significance of these factors, showing that the Time-out procedure, when properly implemented, can markedly reduce surgical adverse events even in resource-constrained countries or settings (6, 7). For instance, recent studies conducted in Africa and Southeast Asia have demonstrated that standardized implementation of the Time-out procedure significantly enhances surgical safety and increases healthcare providers’ safety awareness, despite limited resources (6, 7). However, these studies also emphasize that local cultural contexts, institutional management structures, and resource allocation profoundly influence implementation outcomes. These influencing factors may similarly exist in the Chinese context, yet have received insufficient attention.

To address these critical gaps, the present study investigates both the awareness and actual implementation of the Time-out procedure among surgeons, anesthesiologists, and operating room nurses in China. By analyzing the key factors influencing these aspects, this research aims to provide actionable clinical insights, guiding healthcare managers toward developing targeted interventions to improve surgical safety. This study aims to: (1) assess surgical personnel’s awareness and practical implementation of the preoperative time-out protocol; (2) identify demographic, professional, managerial and cultural factors affecting its cognition and execution; (3) offer evidence-based references for targeted training and management optimization, so as to standardize clinical practice, improve surgical safety and reduce preventable adverse events.

## 2 Objects and methods

### 2.1 Subject investigated

From March to June 2024, a total of 328 surgical team members were recruited from the operating rooms of four tertiary hospitals in Zhongshan City using convenience sampling. The sample size was calculated based on the requirements of multiple linear regression analysis, which recommends at least 10 participants per independent variable (8). Given that 12 independent variables were considered (age, gender, working years, professional title, education, occupation, work shift, participation in Time-out procedure training, initiator’s professional title, completeness of the Time-out procedure, supervision and management systems, and hospital safety atmosphere), a minimum sample size of 144 participants was required after accounting for a 20% non-response rate. The 328 completed questionnaires exceeded this requirement, ensuring adequate statistical power for the analyses.

The inclusion criteria were: (1) surgeons, anesthesiologists, and operating room nurses holding valid practicing qualifications; (2) at least one year of frontline clinical experience in grade A tertiary hospitals; and (3) voluntary participation with informed consent. The exclusion criteria included: (1) individuals engaged in further studies, non-clinical tasks, or reemployment; and (2) staff unavailable due to illness or other commitments during the study period.

Although convenience sampling facilitated the data collection process, it may introduce selection bias, potentially limiting the generalizability of the findings. Specifically, the current results may primarily reflect conditions within tertiary hospitals in Zhongshan City, restricting their applicability to hospitals in other regions or of different levels. Future research is recommended to utilize stratified random sampling to enhance sample representativeness and to verify the applicability of these findings across broader healthcare settings. The study protocol was reviewed and approved by the Ethics Committee of Zhongshan City People’s Hospital. (NO: K2022-243). Prior to questionnaire distribution, all participants provided informed consent electronically. The consent statement clearly described the study purpose, survey procedures, estimated completion time, data confidentiality measures, and the voluntary nature of participation. All participants were informed that they could withdraw at any time without penalty before submitting the questionnaire.

### 2.2 Study type and design

*Study type*: Cross-sectional descriptive and analytical study.

*Study design*: A cross-sectional online questionnaire survey was conducted in four tertiary hospitals.

*Data normality*: The data were tested for normality before parametric analysis. The continuous variables conformed to a normal distribution; therefore, *t*-test, one-way ANOVA, Pearson correlation, and stepwise multiple linear regression were used for statistical analysis.

#### 2.2.1 Research tool

1. A general data questionnaire was formulated by the researchers to collect basic demographic and professional information, including age, gender, working years, title, education, occupation, position, working period (day and night shift), participation in Time-out procedure team training, Time-out procedure initiator title, and involvement in the complete Time-out process with its corresponding supervision and management system. The “training” variable captures self-reported prior exposure to any form of Time-out training (e.g., workshops, online modules, on-the-job instruction), not a standardized intervention.
2. A self-developed questionnaire was used to assess both the awareness and implementation of the Time-out procedure. Its development was based on relevant literature (2, 3, 5, 9, 10). The preliminary draft of the questionnaire was developed and subsequently revised based on interviews with 10 surgical team members (4 operating room nurses, 3 anesthesiologists, and 3 surgeons), evaluations during an expert meeting (involving 6 surgical medical experts, 2 operating room nursing experts, and 2 anesthesiology experts with at least a master’s degree and deputy senior or higher titles), and the results of a pre-experiment survey conducted among 30 eligible surgical team members. The final version of the questionnaire comprised two sections with a total of 26 items. “Section 1, the Time-out procedure cognition questionnaire, covers five domains: (a) definition and execution timing (2 items), (b) executor (3 items), (c) procedure content (15 items), (d) function and significance of execution (3 items), and (e) compliance requirements (3 items). Each item was rated on a 5-point Likert scale, ranging from 1 (“very ignorant”) to 5 (“very knowledgeable”), yielding a total score range of 26–130. A total score of 26–130, the higher the assessment score, the higher the cognitive level of the surgical team members regarding the Time-out procedure. Scores below 45 were considered indicative of low awareness, 45–90 moderate, and above 90 high. Section 2, the Time-out Procedure Execution Questionnaire, was designed to more accurately assess the actual execution of the Time-out procedure by the surgical team and identify specific difficulties encountered. It was revised as follows: (a) Frequency-based Design: The original yes/no question “Do you perform the Time-out procedure for every surgery?” was replaced with a frequency-based format with the following response options: 1 = Never, 2 = Rarely, 3 = Sometimes, 4 = Often, and 5 = Always. This approach provides a more nuanced measure of execution frequency. (b) Inclusion of Open-ended Questions: To gain deeper insights into the barriers and challenges associated with executing the Time-out procedure, the questionnaire incorporates open-ended questions such as: “Please describe the main difficulties or obstacles you encounter when performing the Time-out procedure” and “What improvements do you suggest to better ensure patient safety during the Time-out procedure?” Responses were analyzed using content analysis to identify key themes. (c) Dichotomous Scoring Revision: For items retaining a yes/no format, scoring was clarified as 0 for “no” and 1 for “yes,” preserving the binary data structure. These revisions were implemented to enhance the accuracy of the questionnaire in reflecting the actual execution of the Time-out procedure and to capture detailed insights into the challenges encountered and areas for potential improvement. The content validity index of the questionnaire items ranged from 0.81 to 1.00, and the overall Cronbach’s *α* coefficient was 0.817. It is acknowledged that the self-developed questionnaire, despite undergoing expert review and pre-survey validation, may have limitations in terms of its coverage of the multidimensional aspects of surgical team behavior.” Furthermore, as the questionnaire relies on self-reported data, there is an inherent risk of response bias due to social desirability or time constraints.
3. The evaluation of the operating room safety culture atmosphere was conducted using a scale originally developed by Gershon et al. (11), and later adapted into Chinese by Xu Na et al. (12). This scale, along with a self-developed operating room safety culture atmosphere assessment scale, was used to evaluate the perceptions of surgical team members. The scale comprises five domains: management and support for operating room safety (5 items), obstacles to safety work implementation (4 items), safety reporting systems and training (5 items), team conflict and communication (3 items), and job responsibilities and systems (3 items). All 20 items were rated on a 5-point Likert scale (1 = “very disagree” to 5 = “very agree”), yielding a total score range of 20–100, with higher scores indicating a more favorable safety climate. In a preliminary investigation involving 30 surgical team members, this scale demonstrated good reliability and validity, with a Cronbach’s *α* coefficient of 0.809 and a content validity of 0.91.

### 2.3 Time-out procedure knowledge test

To objectively assess the surgical team’s mastery of the key steps and essential components of the time-out procedure, an additional section—“Time-out Procedure Knowledge Test”—was incorporated into the questionnaire. This section is designed to evaluate factual knowledge, complementing the self-reported measures of awareness and adherence.

Design and scoring: multiple-choice questions.

*Question 1*: “What is the primary purpose of the time-out procedure?”

a) Prevent surgical errors

b) Arrange team roles

c) Confirm post-operative care

d) Allocate surgical resources.

Correct answer: a) Prevent surgical errors.

*Question 2*: “Which of the following is NOT a component of the time-out procedure?”

a) Verifying the surgical site

b) Confirming patient identity

c) Discussing the schedule for the next patient

d) Confirming the surgical procedure.

Correct answer: c) Discussing the schedule for the next patient.

*Question 3*: “Which information must be verified during the time-out procedure?”

a) Patient identity

b) Surgical site

c) Type of surgery

d) Doctor’s shift schedule.

Correct answer: a), b), and c) (This can be presented as a multiple-answer question to ensure that the respondents recognize all required elements).

True/False Questions.

*Question 4*: “The time-out procedure must be performed before every invasive surgery.”

Correct answer: True.

*Question 5*: “The time-out procedure requires active participation from all members of the surgical team.”

Correct answer: True.

Each correctly answered item is awarded one point, while an incorrect or omitted answer receives zero points. The cumulative score reflects the respondent’s level of factual knowledge regarding the time-out procedure.

*Optional qualitative addendum*: To further explore respondents’ perceptions, an open-ended question may be added: “Please briefly describe the role of the time-out procedure in ensuring surgical safety and identify its most critical component.”

### 2.4 Data collection method

#### 2.4.1 Recruitment and training of data collectors

Four registered nurses working in operating rooms were recruited as data collectors. Each received 2 hours of standardized training delivered by the research team, covering: (a) the purpose and background of the study, (b) the content and scoring rules of each questionnaire section, (c) ethical considerations (informed consent, confidentiality, anonymity), and (d) procedures for distributing online questionnaires, responding to participant inquiries, and screening invalid responses. After training, each data collector passed a competency assessment before being authorized to collect data.

#### 2.4.2 Participant recruitment and questionnaire distribution

Between March and June 2024, the research team contacted the head nurses and operating room supervisors of the four participating tertiary hospitals in Zhongshan City to explain the study’s purpose and significance, thereby securing their consent and cooperation. The four trained data collectors then visited the operating rooms of each hospital. Under conditions of confidentiality and anonymity, they provided a standardized verbal explanation of the study to all eligible surgical team members (surgeons, anesthesiologists, and operating room nurses), obtained informed consent, and emphasized the voluntary nature of participation. Following this, the data collectors issued questionnaire QR codes and web links (hosted on the Questionnaire Star platform, Changsha, China) in the operating rooms of the four grade A tertiary hospitals.

#### 2.4.3 Online survey administration and quality control

The online questionnaire was configured with the following settings to ensure data integrity: all items were required before submission; participants were advised to complete the questionnaire within 15–20 min, and the platform automatically recorded completion time; each IP address and device could submit only one response. An attention-check item was embedded to detect random responding. During the data cleaning process, questionnaires completed in under 5 minutes were excluded. Once collected, the data collectors cross-checked the responses and excluded invalid questionnaires in accordance with the above criteria. A total of 328 questionnaires were collected, and all collected questionnaires met the validity criteria, yielding an effective response rate of 100%.

### 2.5 Statistical methods

Raw data were exported from the online questionnaire platform, checked for completeness, and transferred into Excel for initial sorting. Subsequent statistical analyses were conducted using SPSS version 26.0 (IBM Corp., Armonk, NY, United States). Statistical significance was set at *α* = 0.05. Descriptive analyses were performed to summarize the frequency-based questionnaire items (means, standard deviations, and frequency distributions), which reflected the overall implementation rate of the Time-out procedure. Open-ended responses were analyzed using qualitative content analysis to categorize and summarize main challenges and proposed improvements. Dichotomous items were scored as 0 (“no”) or 1 (“yes”) and analyzed separately from scaled data.

Inferential statistical tests included t-tests, one-way analysis of variance (ANOVA), Pearson’s correlation analysis, and stepwise multiple linear regression analyses. Given the inclusion of 12 independent variables in the regression models, Multicollinearity was assessed using Variance Inflation Factors (VIF), with all values falling below the threshold of 5.0, indicating no significant collinearity issues. Residual plots and statistical tests confirmed that the assumptions of normality and independence were met. Model fit was evaluated using the adjusted coefficient of determination (*R*^2^).

Additionally, sensitivity analyses were performed to assess the robustness of regression results. After removing potential outliers and adjusting key combinations of variables, the main conclusions remained consistent, confirming the stability and reliability of the findings.

## 3 Results

The score of the Time-out procedure knowledge test was 3.80 ± 0.90, suggesting that surgical team members possessed moderate relevant professional knowledge. On a scale score of the Time-out procedure cognition, execution, and operating room safety culture atmosphere, 328 surgical team members scored 72.95 ± 14.82. The evaluation score of the operating room was 68.83 ± 14.76, and the Time-out procedure execution score was 2.95 ± 0.41. In the item “Did each operation you performed Time-out,” 149 (45.43%) surgical team members answered “yes.” In the Entry item “Time-out procedure when executed, whether the three parties arrive,” 127 (38.72%) members of the surgical team answered “yes.” Additionally, in the entry item “Time-out procedure when executed, did the three parties stop working to cooperate?” Of the surgical team members, 101 (30.79%) answered “yes.” The implementation score reflected the self-reported frequency of performing the time-out procedure rather than an objective assessment of procedural quality. Detailed scoring information is depicted in Table 1.

**Table 1:** Scores of surgical team members regarding time-out procedure awareness, execution, and operating room safety culture (mean ± standard deviation; all dimension scores based on a 5-point likert scale).

Project	Total points	The entries are equally divided
Time-out procedure cognition	72.95 ± 14.82	3.31 ± 0.85
Time-out procedure definition and execution timing	6.76 ± 0.61	3.38 ± 0.59
Time-out procedure executor	9.86 ± 1.79	3.29 ± 0.64
Time-out procedure execution content	39.51 ± 6.50	2.93 ± 0.58
Time-out Role and significance of program execution	9.01 ± 1.20	3.00 ± 0.60
Time-out procedure implementation of the standard requirements	8.90 ± 0.97	2.96 ± 0.59
Time-out procedure execution	2.95 ± 0.41	0.59 ± 0.50
Operating room safety culture and atmosphere assessment	68.83 ± 14.76	3.44 ± 0.80
Management and support of operating room safety	16.90 ± 3.91	3.46 ± 0.81
Barriers to the execution of the operating room safety work	12.80 ± 2.56	3.20 ± 0.79
Operating room safety reporting system and training	18.36 ± 4.02	3.66 ± 0.90
Conflict and communication of the team	10.05 ± 1.31	3.35 ± 0.80
Job responsibilities and system of team members	9.85 ± 1.26	3.28 ± 0.78

Comparison of the cognitive scores of different working years, professional titles, academic qualifications, occupations, and the surgical team members participating in the Time-out procedure team training. The difference was statistically significant (*p* < 0.05). Comparison of different occupations, working years, professional titles, working periods (day and night shift), participation in Time-out procedure team training, Time-out procedure initiator title, perfect Time-out procedure process and supervision and management system, and the scores of Time-out procedure execution by surgical team members (*p* < 0.05), are depicted in Table 2.

**Table 2:** Comparison of scores for awareness and execution of the Time-out procedure among surgical team members with different demographic and occupational characteristics (mean ± standard deviation; analyzed using *t*-test or one-way ANOVA).

Feature	Project	*N*	Cognitive score	Statistics	*p* price	Executing the score	Statistics	*p* price
Age (year)	24 ~ 30	85	71.58 ± 14.69	0.435	0.648 b	2.89 ± 0.43	1.095	0.336 b
	>30 ~ 40	137	72.90 ± 14.78			2.94 ± 0.40		
	>40	106	73.57 ± 14.90			2.98 ± 0.43		
Years of service (years)	1 ~ 10	106	69.55 ± 13.79	3.412	0.034 b	3.07 ± 0.50	12.360	0.000 b
	>10 ~ 20	158	73.28 ± 14.81			2.97 ± 0.43		
	>20	64	75.06 ± 14.90			2.72 ± 0.40		
Sex	Man	133	72.90 ± 14.83	0.042	0.967 a	2.96 ± 0.40	−0.432	0.666 a
	Woman	195	72.97 ± 14.81			2.94 ± 0.42		
Professional ranks and titles	Elementary	111,1	69.99 ± 14.90	4.192	0.016 b	3.15 ± 0.45	14.760	0.000 b
	Middle rank	28	73.24 ± 14.85			2.93 ± 0.42		
	Senior	89	76.07 ± 14.79			2.84 ± 0.39		
Record of formal schooling	Doctor	180	74.89 ± 15.21	3.082	0.047 b	2.94 ± 0.40	0.040	0.996 b
	Master	107	72.83 ± 14.80			2.95 ± 0.42		
	Undergraduate course	41	68.56 ± 14.91			2.93 ± 0.42		
Occupation	The surgeon	107	69.73 ± 14.76	5.068	0.007 b	2.80 ± 0.35	17.880	0.000 b
	Anesthesiologist	95	71.05 ± 14.85			2.89 ± 0.42		
	Theatre nurse	126	75.87 ± 15.44			3.24 ± 0.50		
Working hours	Day shift	217	72.97 ± 14.84	−0.035	9.72 a	3.21 ± 0.48	−7.969	0.000 a
	Late night	111	72.91 ± 14.80			2.79 ± 0.39		
	Shift							
Participating in team training	Yes	107	79.89 ± 15.27	−7.347	0.000 a	3.11 ± 0.55	−8.667	0.000 a
	Deny	221	67.47 ± 13.89			2.67 ± 0.36		
Time-out procedure of the initiator of the professional title	Elementary	29	72.90 ± 14.81	1.021	0.997 b	2.74 ± 0.38	26.200	0.000 b
	Middle rank	44	72.95 ± 14.83			2.83 ± 0.47		
	Senior	34	72.94 ± 14.82			3.48 ± 0.52		
Whether there is a perfect process and supervision system	Yes	109,	78.93 ± 15.27	−6.352	0.000 a	3.20 ± 0.39	−8.851	0.000 a
	Deny	219	67.94 ± 14.50			2.76 ± 0.44		

To further evaluate the impact of training on Time-out procedure execution, a t-test was performed comparing execution scores between trained and untrained surgical team members. The results indicated that the trained group (3.11 ± 0.55) scored significantly higher than the untrained group (2.67 ± 0.36), with a statistically significant difference (*t* = 8.667, *p* < 0.001).

Correlation between Time-out cognition, execution, and operating room safety culture. There was a positive correlation between the cognition and execution scores for the Time-out procedure and operating room safety culture scores among surgical team members (*r* = 0.391, 0.504; *p* < 0.001).

Multiple linear regression analysis of the cognition and execution of the surgical team members on Time-out procedures as the dependent variable. Working years (> 10–20 years as control), professional title (with intermediate title as comparison), educational background (master’s degree as comparison), occupation (setting dumb variables with anesthesiologist as control), participation in Time-out procedure team training (1 = yes, 2 = no),and six variables of the total score of operating room safety culture atmosphere (original value input) were used as independent variables for multiple linear gradual regression analysis (*α* entry = 0.05, *α* Out = 0.10). The results indicated that occupation, participation in Time-out procedure team training, and safety culture in the operating room are the main factors affecting the perception of surgical team members on Time-out procedure (*p* < 0.05); 44.9% of the total explained variation is depicted in Table 3 for more details. Performing the Time-out procedure by the surgical team members, independent variables included working years (> 10–20 years as reference variables), title (with an intermediate title as reference variables), working period (1 = day class, 2 = late night shift), occupation (with the anesthesiologist), participation in Time-out procedure team training (1 = yes, 2 = no), the title of Time-out procedure initiator (with an intermediate title as the control setting mute variable), a perfect Time-out procedure process and supervision and management system (1 = yes, 2 = no), total score of operating room safety culture atmosphere (original value entry), and eight variables as independent variables for multiple linear gradual regression analysis (*α* entry = 0.05; α Out = 0.10). The results revealed that occupation, working period, title of Time-out procedure initiator, perfect Time-out procedure process and supervision and management system, and safety atmosphere of the operating room were the main factors affecting the implementation rate of Time-out procedure by surgical team members (*p* < 0.05), 47.1% of the total explained variation are indicated Table 4 for more details.

**Table 3:** multiple linear regression analysis of time-out procedure (*n* = 328).

Argument	*β*price	SE price	*β*price	*t*price	*p price*
Constant term	31.680	2.872	—	11.031	<0.001
Occupation					
Theatre nurse	1.497	0.358	0.207	4.182	0.006
Participation in the Time-out procedure team training	2.048	0.371	0.277	5.520	0.003
Operating room safety culture atmosphere	0.869	0.112	0.197	7.7591	0.000

*R*^2^ = 0.472, adjusted *R*^2^ = 0.449, *F* = 32.790, *p* < 0.001.

**Table 4:** Multiple linear regression analysis of time-out procedure (*n* = 328).

Argument	*β* price	SE price	*β* price	tprice	*p*price
Constant term	31.692	2.267	—	13.980	<0.001
Occupation					
Theatre nurse	0.987	0.201	0.093	4.910	0.009
Operating room safety culture atmosphere	1.829	0.339	0.173	5.395	0.008
Working hours	0.075	0.012	0.204	6.250	0.006
Time-out procedure of the initiator of the professional title					
Senior professional title	1.083	0.218	0.347	4.968	0.009
Perfect time-out procedure process and supervision system	0.983	0.076	0.272	12.934	<0.001

*R*^2^ = 0.504, adjusted *R*^2^ = 0.471, *F* = 26.983, *p* < 0.001.

## 4 Discussion

### 4.1 Surgical team members have a moderate level of cognition of the time-out procedure, with low execution score and rate

This study indicated that the 328 surgical team members achieved a Time-out procedure cognition score of 72.95 ± 14.82, placing them within the moderate range of 45–90. This finding is consistent with the results reported by Guo Yan (2) and Zhang Lixin (13), suggesting that overall awareness remains suboptimal. One contributing factor may be that, although the Time-out procedure was officially incorporated into China’s surgical verification system in 2010, its promotion and standardized training in many domestic hospitals have been limited. Additionally, the routine verification process in domestic settings is typically conducted by three parties, and discrepancies in understanding among these team members may further compromise standardized practice (3).

Moreover, the Time-out procedure execution score was only 2.95 ± 0.41, with only 149 (45.43%) respondents affirming routine implementation. Similar low execution rates were observed in studies by Gong et al. (14) but were lower than those reported in another study (15). Furthermore, only 127 (38.72%) and 101 (30.79%) members, respectively, indicated complete adherence to the procedure, underscoring issues of low execution rate, non-standard implementation, and lower pass rates. These findings likely reflect the fact that surgeons and anesthesiologists in many hospitals in China have not received standardized Time-out procedure training, leading to variations in understanding and practice.

Compared with international studies, the execution rate observed in this study is lower than reports from developed countries in Europe and North America (15). This disparity may be attributable to cultural factors, such as the prevalent hierarchical structures in Chinese hospitals and the predominantly unidirectional (rather than interactive) communication among healthcare professionals, which can hinder effective teamwork during the Time-out process. Additionally, some healthcare providers perceive the procedure as a delay to the surgical workflow, a misconception that may further inhibit standardized execution. Consequently, adapting the procedure to the local cultural context and optimizing management strategies appear to be critical steps in improving the efficiency of Time-out implementation.

To enhance the applicability and effectiveness of the Time-out procedure, it is recommended that hospital managers not only intensify surgical safety verification and educational efforts but also foster a stronger sense of accountability among team members by strengthening audit mechanisms. Such measures could promote consistency and standardization in the execution of the Time-out procedure, thereby enhancing patient safety and reducing surgical risks.

### 4.2 Analysis of the cognitive factors influencing the time-out procedure by the surgical team members

The results of this survey indicate that occupation, whether to participate in Time-out team training and operating room safety culture are the main factors affecting the cognition of Time-out team members, which includes operating room nurses, participating in Time-out procedure team training and operating room safety culture of a high level of Time-out team members cognition (*p* < 0.05), which is similar to Guo Yan (2) and Zhang Xia (16) research results. Furthermore, working years, professional title, educational background, and institutional supervision system also significantly affected surgical team members’cognitive level of the Time-out procedure (all *p*<0.05). The reason was investigated because the operation involved multiple disciplines, multiple team cooperation, higher operating room adverse events occurrence probability, and the Time-out procedure’s main purpose was to promote surgical safety, ensure the correct surgical patients, correct operation site, and correct operation way (17, 18). This was to ensure the operating room’s safety and reduce the powerful tool of adverse events. Staff with longer working years and higher professional titles have long participated in perioperative clinical work. They have accumulated abundant practical experience and established standard understanding of surgical safety verification specifications. Staff with higher educational background have accepted systematic professional study and safety training, and thus have better cognition of Time-out related knowledge. Complete operating room procedure specifications and sound supervision and management systems can standardize staff’s daily safety behavior, enhance their safety awareness, and further elevate their overall cognition level of Time-out procedure. The verification work of operating rooms in most domestic hospitals is undertaken by nurses, and the promotion and application of Time-out procedures can reduce the verification pressure on nurses. As direct beneficiaries, surgical nurses hope to vigorously promote Time-out procedures and pay more attention to the mastery of relevant knowledge to facilitate the promotion to surgeons and anesthesiologists. Studies have confirmed that education and training are key to successfully applying the Time-out procedure (19). Ezzi et al. (20) introduced the Time-out procedure into the course of medical students. They found that medical students were highly aware of the Time-out procedure and performed well during the anatomy course. Campbell et al. (21) also confirmed that education on Time-out procedures for the surgical team can effectively improve the cognitive level and execution level of these procedures among team members. It is suggested that participation in Time-out procedure team training can improve the level of Time-out procedure cognition and executive confidence of surgical team members. Safety atmosphere refers to the overall perception of the organization’s environment and describes the individual cognition of work environment safety value (22). When the surgical team’s perception of the operating room safety culture level is high, the safety culture consciousness increases, and they will be more willing to prevent surgical risk events and actively take preventive measures to mitigate operation risk accidents. They will actively understand safety verification content and specification verification behavior to improve the Time-out procedure cognition. It suggests that hospital managers should pay more attention to the Time-out of surgeons and anesthesiologists. For the cognition of the Time-out procedure, they develop the surgical safety verification training program, provide the surgical team members with simulation verification and Time-out procedure scenario training, provide theoretical training of surgical safety verification and Time-out procedure in the form of the workshop, and improve the surgical team of Time-out procedure, which actively drive the surgical team to establish the safety culture concept of prevention and control of adverse events, strengthen the safety education, strengthen the safety concept, and create the surgical safety culture atmosphere (3).

### 4.3 Analysis of the factors influencing the performance of the time-out procedures by the surgical team members

The results of this survey reveal that occupation, working period, title of Time-out procedure starter, perfect Time-out procedure process and supervision management system, and safety atmosphere of the operating room are the main factors affecting the implementation rate of Time-out procedure by the surgical team members, surgeon, late night shift, junior high school Time-out procedure starter, lack of perfect Time-out procedure and supervision and management system, low level of the operating room safety atmosphere, and Time-out low level of procedure execution (*p* < 0.05). This is similar to the results of Guo Yan et al. (2) and Etheridge et al. (23). Although the knowledge test score reached 76.0% (3.80 ± 0.90), the actual implementation rate was only 45.43%. This “knowledge–behavior gap” suggests that the barriers to Time-out compliance in Zhongshan hospitals may not be due to a lack of awareness, but rather stem from systemic or cultural factors (such as hierarchy or workflow issues) that prevent the application of existing knowledge. Analysis reasons: Due to the serious shortage of human resources of doctors in most hospitals in China, the surgeon not only needs to consider the safety of the patient, ward rounds, and the treatment of clinical things but also the outpatient work and the completion of the surgery. After completing their ward work, many surgical surgeons feel more concerned about the progress of the operation when coming to the operating room. They thought that the patient situation was already very well understood and already checked before anesthesia, viewed stopping surgery from participating in the Time-out procedure as a waste of time, were unwilling to cooperate with stopping the operation for verification, and just a formality or a lower physician or assistant to complete the Time-out procedure. The late-night shift surgery team is even more short of manpower, and patients are severe after the night shift operation. A race against time is necessary to save the patients; this leads to the implementation of routine surgical procedures. It will also associated with surgical safety verification, and time-out procedures implementation will not be timely and will be non-standard. The existence of traditional occupational grades in medical institutions and the differences in discipline status in different specialties hindered teamwork communication (2) and research findings. When the senior surgeon and the anesthesiologist initiate the Time-out procedure, the procedure in the operating room more easily initiates the implementation of the study (4). This study investigated the Time-out procedure execution and found that the higher the professional title, the higher the academic status of the hospital, and the higher the program execution. It also confirms this point: A complete Time-out procedure allows members of the surgical team to perform the Time-out procedure; the supervision and management system can urge the surgical team to standardize the Time-out procedure execution behavior. A safety atmosphere can affect the safety behavior of the employees. Studies have indicated that in the hospital safety atmosphere and medicine, the safety behavior of the service staff is closely related, and the better the safety atmosphere, the more willing the medical staff are to implement safe behavior (22). Accordingly, the higher the operating room members perceive the safe atmosphere of the operating room, the more willing they are to regulate their behavior and improve compliance with surgical safety verification and Time-out procedures. It prompts managers to arrange the workload of surgeons reasonably, pay attention to the training of surgeons’ Time-out procedures, improve their cognition of Time-out procedures, optimize the night shift operation verification process, adopt flexible scheduling, reasonably arrange human resources, establish a sound Time-out procedure and supervision and management system to supervise the implementation of Time-out procedure, and pay attention to develop surgeons with high titles and high academic status as the initiator and advocate of Time-out procedure, and promote the standardization of Time-out procedure.

Finally, it should be noted that the use of convenience sampling and the selection of tertiary hospitals exclusively in Zhongshan City may limit the generalizability of the study’s results to other regions or hospital levels. Future research should consider expanding the scope of investigation to verify the applicability of these findings across different geographical areas and hospital tiers.

## 5 Conclusion

The results showed that surgical team members had moderate cognition regarding the Time-out procedure, yet their execution scores and implementation levels were comparatively low. This study also distinguished different influencing factors for cognition and implementation.

## 6 Limitations

It should be noted that this study enrolled 328 surgical team members from four tertiary hospitals in Zhongshan City to explore their cognition, implementation status and influencing factors of the Time-out procedure. Although this sampling method is feasible in clinical investigation, it may limit the generalizability of the research findings, which can only reflect the practical situation in this local area to a certain extent.

In this study, core influencing factors were selected in accordance with relevant theoretical basis. However, potential deviations may exist in model assumptions, so the results of regression analysis should be interpreted with caution. Moreover, all data regarding Time-out procedure implementation were collected through self-reported questionnaires. We adopted knowledge tests to reduce subjective bias, but direct on-site observation was not performed in this research. Accordingly, the research results are susceptible to social desirability bias, which may lead to overestimation of the actual implementation compliance rate. As this study was exploratory in nature, formal factor analysis was not performed, which is a limitation of the present work.

For further research, stratified random sampling and large-scale multicenter surveys covering different regions are recommended. In addition, combining objective field observation with practical evaluation methods can achieve more accurate assessment on the actual implementation of the Time-out procedure, and further verify the reliability of the present research results.

## Data Availability

The original contributions presented in the study are included in the article/supplementary material, further inquiries can be directed to the corresponding author.
